# Comparative Genomic Analysis Reveals Ecological Differentiation in the Genus *Carnobacterium*

**DOI:** 10.3389/fmicb.2017.00357

**Published:** 2017-03-08

**Authors:** Christelle F. Iskandar, Frédéric Borges, Bernard Taminiau, Georges Daube, Monique Zagorec, Benoît Remenant, Jørgen J. Leisner, Martin A. Hansen, Søren J. Sørensen, Cécile Mangavel, Catherine Cailliez-Grimal, Anne-Marie Revol-Junelles

**Affiliations:** ^1^Laboratoire d’Ingénierie des Biomolécules, École Nationale Supérieure d’Agronomie et des Industries Alimentaires – Université de LorraineVandoeuvre-lès-Nancy, France; ^2^Laboratory of Food Microbiology, Department of Food Science, Fundamental and Applied Research for Animal and Health, University of LiègeLiège, Belgium; ^3^UMR1014 SECALIM, INRA, OnirisNantes, France; ^4^Department of Veterinary Disease Biology, Faculty of Health and Medical Sciences, University of CopenhagenFrederiksberg, Denmark; ^5^Molecular Microbial Ecology Group, University of CopenhagenCopenhagen, Denmark

**Keywords:** lactic acid bacteria, *Carnobacterium*, 16S meta-barcoding, comparative genomic analyses, ecological differentiation

## Abstract

Lactic acid bacteria (LAB) differ in their ability to colonize food and animal-associated habitats: while some species are specialized and colonize a limited number of habitats, other are generalist and are able to colonize multiple animal-linked habitats. In the current study, *Carnobacterium* was used as a model genus to elucidate the genetic basis of these colonization differences. Analyses of 16S rRNA gene meta-barcoding data showed that *C. maltaromaticum* followed by *C. divergens* are the most prevalent species in foods derived from animals (meat, fish, dairy products), and in the gut. According to phylogenetic analyses, these two animal-adapted species belong to one of two deeply branched lineages. The second lineage contains species isolated from habitats where contact with animal is rare. Genome analyses revealed that members of the animal-adapted lineage harbor a larger secretome than members of the other lineage. The predicted cell-surface proteome is highly diversified in *C. maltaromaticum* and *C. divergens* with genes involved in adaptation to the animal milieu such as those encoding biopolymer hydrolytic enzymes, a heme uptake system, and biopolymer-binding adhesins. These species also exhibit genes for gut adaptation and respiration. In contrast, *Carnobacterium* species belonging to the second lineage encode a poorly diversified cell-surface proteome, lack genes for gut adaptation and are unable to respire. These results shed light on the important genomics traits required for adaptation to animal-linked habitats in generalist *Carnobacterium*.

## Introduction

Lactic acid bacteria (LAB) include various genera and many species that have been investigated for decades because of their major role in food fermentations and their health benefit potential as probiotics. Understanding how LAB fitness can increase through evolution in these various habitats is critical for exploiting their beneficial properties. LAB are well-known for their ability to colonize animal-derived food, i.e., meat, fish, and dairy products, and for being members of the gastrointestinal (GI) tract and the vagina microbiota (Douglas and Klaenhammer, 2010; Douillard and de Vos, 2014). LAB can be divided into specialized and generalist bacteria. Typically, the specialists that are used as starter cultures for a narrow range of fermented products are characterized by a low genetic diversity (Delorme et al., 2010). Their genomes exhibit signs of massive losses of genes involved in biosynthetic pathways (Douglas and Klaenhammer, 2010). To compensate the loss of these functions, genes encoding transporters for amino acids or carbohydrates were gained to allow growth in nutritional rich fermentation environments (Lorca et al., 2007). These genomic changes were accompanied by a specialization to food matrices, particularly exemplified in dairy strains. Other LAB also exhibit genomes characteristic of their ecological specialization. *Lactobacillus iners* is described to solely colonize the vaginal cavity and harbors one of the smallest LAB genomes presumably because its niche specialization allowed a substantial genome reduction (Macklaim et al., 2011; Mendes-Soares et al., 2014). Another example is the GI tract symbiont *Lactobacillus reuteri*, which is characterized by different lineages each one being apparently adapted to one particular vertebrate host: rodent or human (Frese et al., 2011). By contrast, some species are more generalist in their ability to colonize various environments and are therefore ubiquitous. Species from the genus *Enterococcus* can be found in the GI tract of animals and in a multitude of fermented foods (Santagati et al., 2012). Their adaptation to various environments is strongly linked to the presence in their genome of DNA acquired through horizontal gene transfer (HGT), resulting in a large pan genome (Santagati et al., 2012). Similarly, the genomes of some strains of the ubiquitous species *Lactobacillus rhamnosus* encode multiple lifestyle traits allowing them to reside in diverse habitats (Douillard et al., 2013). However, the interconnection between the ecology of these bacteria and their genomics is not fully understood and need further investigation. Importantly, the genomic traits responsible for adaptation to multiple animal-associated habitats are not clearly defined.

The LAB genera *Enterococcus, Lactobacillus, Lactococcus*, and *Streptococcus* have been intensively investigated, but lately, other genera including *Carnobacterium* have also drawn attention since 16S meta-barcoding studies have shown their significance in food (Chaillou et al., 2015; Duan et al., 2016; Fougy et al., 2016; Jääskeläinen et al., 2016). The genus *Carnobacterium* encompasses 11 species that have been isolated from cold and temperate environments, and from the GI tract of animals as well as from foods of animal origin such as seafood, meat, and dairy products. They are mesophilic and some species are psychrotolerant and able to grow down to 0°C. Some are halotolerant and able to grow with 8% NaCl and some are alkaliphilic with growth up to pH 9.5 (Cailliez-Grimal et al., 2014; Pikuta, 2014; Pikuta and Hoover, 2014). These traits could explain their wide distribution. However, it is apparent that some heterogeneity exists within the genus regarding habitat associations. Some species can be isolated from environments where contact with animals is likely rare as exemplified by *Carnobacterium inhibens* subsp. *gilichinskyi* WN1359, *Carnobacterium* sp. 17-4, and *Carnobacterium* AT7 which were isolated from Siberian permafrost, sea-ice from permanently cold fjords of the Arctic Ocean, and an oceanic trench, respectively (Lauro et al., 2007; Voget et al., 2011; Leonard et al., 2013; Nicholson et al., 2015). Other species, including *C. maltaromaticum* and *C. divergens*, have been found in animal-associated habitats. These two species are the most frequently isolated carnobacteria from various sources (Leisner et al., 2007) and belong to the dominant bacterial communities in meat and fish derived food (Chaillou et al., 2015; Duan et al., 2016; Fougy et al., 2016; Jääskeläinen et al., 2016). These bacteria are therefore very interesting models to investigate the genomic traits responsible for adaptation to animal-associated habitats. In the genus *Carnobacterium*, the genome size ranges from 2.4 Mbp for *C. inhibens* subsp. *gilichinskyi* WN1359 (Leonard et al., 2013), to 3.7 Mbp for *C. maltaromaticum* (Cailliez-Grimal et al., 2013). It has been suggested that the larger genome of the latter species is the basis of its success in colonizing various habitats (Leisner et al., 2007). However, it appears that genome size is not necessarily a predictor of the ability to occupy diverse habitats since the genome of *C. divergens* strains is relatively small (∼2.7 Mbp), but this species is still able to colonize various habitats (Sun et al., 2015; Remenant et al., 2016).

The aim of this study was to investigate the ecological niches *Carnobacterium* species can occupy and to identify the genomic traits responsible for the high ecological success of some *Carnobacterium* species and the possible underlying adaptive mechanisms. For that purpose, we analyzed the relative abundance of *Carnobacterium* species using 16S rDNA metagenomic data. Subsequently, we compared the genomes of *Carnobacterium* strains isolated from different environments (mainly from cold aquatic habitats), and animal-associated habitats (live animal and foods).

## Materials and Methods

### 16S Meta-Barcoding Sequence Analysis

The data obtained from 681 samples of various ecological origins were analyzed with a focus on *Carnobacterium*. A database of V1–V3 16S rRNA gene sequence datasets available at the FARAH Institute (University of Liège, Belgium) was used to delineate the species composition of the *Carnobacterium* genus within four types of matrices: food products, animal samples, human and animal feces and environment. This database was built from 2010 to 2015 by merging the data obtained from several single projects hosted at the FARAH Institute. The datasets were produced as previously described (Rodriguez et al., 2015) by sequencing the V1–V3 16S rDNA hypervariable region with an MiSeq sequencer using v3 reagents (ILLUMINA, USA).

Sequence read processing was employed as previously described (Rodriguez et al., 2015) using the MOTHUR software package v1.35 (Schloss and Handelsman, 2003) and the UCHIME algorithm (Edgar et al., 2011) for alignment and OTU clustering (distance 0.03) and chimera detection, respectively. 16S gene sequence reference alignment and taxonomical assignation were based upon the SILVA database (v1.15) of full-length 16S rDNA sequences.

For each sample, Operational Taxonomic Units (OTUs) belonging to the *Carnobacterium* genus were extracted. Corresponding reads were further assigned to *Carnobacterium* species using a local BLASTn algorithm vs. the SILVA database (v1.15). Reads were assigned to a defined species when identical to the best hit (four mismatches were allowed).

Statistical differences of the different species proportion inside each type of matrix were assessed with non-parametric Kruskal–wallis test with Dunn’s *post hoc* tests using PRISM6 (GraphPad Software).

### Carnobacterium Genome Analysis

The *Carnobacterium* genome sequences available at the start of this study included five *C. maltaromaticum*, one *C. inhibens* subsp. *gilichinskyi*, one *C. divergens*, and two *Carnobacterium* sp. genomes among which three were complete (*C. maltaromaticum* LMA28, *Carnobacterium* sp. 17.4, and *C. inhibens* subsp. *gilichinskyi* WN1359). The strains originated from different ecological habitats, some from animal-derived food such as the *C. maltaromaticum* strains and *C. divergens* V41, and others from environmental samples (**Table 1**).

**Table 1:** Characteristics of *Carnobacterium* strains.

Species	Strain name	Origin and reference	Genome reference	Accession number
*C. divergens*	V41	Fish viscera (Pilet et al., 1994)	Remenant et al., 2016	FLLU01000001 to FLLU01000032
*C. inhibens* subsp. *gilichinskyi*	WN1359	Siberian permafrost (Leonard et al., 2013)	Leonard et al., 2013	CP006812 to CP006817
*C. maltaromaticum*	ATCC35586^∗^	Diseased trout (Hiu et al., 1984)	Leisner et al., 2012	NZ_AGNS00000000.1
	LMA28^∗^	Soft ripened cheese (Millière et al., 1994)	Cailliez-Grimal et al., 2013	HE999757.2
	DSM20342 MX5^∗^	Milk with malty flavor (Miller et al., 1974)		NZ_JQMX00000000.1
	3.18^∗^	Pork meat product (Laursen et al., 2005)	Iskandar et al., 2016	PRJEB8756
	ML.1.97^∗^	Fresh salmon (Laursen et al., 2005)	Iskandar et al., 2016	PRJEB9002
*Carnobacterium* sp.	AT7	Aleutian trench (Lauro et al., 2007)	Lauro et al., 2007	NZ_ABHH00000000.1
	17.4	Cold seawater (Voget et al., 2011)	Voget et al., 2011	NC_015390.1, NC_015391.1

*^∗^Supplementary Figure S1.*

The genome sequences were integrated in the MicroScope platform (Vallenet et al., 2013) to perform automatic and expert annotation of the genes, as well as comparative analysis and secretome prediction by using the integrated SignalP software (Petersen et al., 2011). The gene phyloprofile tool interface was used for searching common or specific genes/regions between a query genome and other genomes or replicons chosen from the ones available in Prokaryotic Genome DataBase (PkGDB; i.e., (re)annotation of bacterial genomes) or complete proteome downloaded from the RefSeq/WGS sections.

The pan/core genome was calculated using two methods. The pan/core genome tool accessible in the comparative genomics section was used with MICFAM parameters of 50 or 80% amino acid (aa) sequence identity and 80% coverage. The Phyloprofile tool from the MicroScope platform was used with a cut-off of 70% aa identity and 80% coverage with the best Bidirectional Best Hit (BBH).

Synteny, defined as an orthologous gene set having the same local organization in species A and B, was determined as sequence similarity by BlastP BBH with at least 30% identity on 80% of the shortest sequence (minLrap 0.8) analyses and co-localization. Metabolic pathways were predicted using the Kyoto Encyclopedia of Genes and the Genomes (KEGG) resources (Kanehisa and Goto, 2000; Kanehisa et al., 2014) and the MetaCyc database (Caspi et al., 2014). A percentage of 70% minimum identity was used to detect the specific and common genes for *C. maltaromaticum*, excluding genes with 30% minimum identity with the four other *Carnobacterium* strains.

Neighbor-joining-based phylogenetic reconstruction was based on the nucleic acid sequence of 10 housekeeping genes (*dnaK, gyrA, polA, lepA, dnaB, gyrB, secA, ftsZ, recG, ileS*) and was performed using MEGA6 by using the Kimura two-parameter model, including transitions and transversions. The candidate tree was tested with 1,000 bootstrap replications (Tamura et al., 2013). The Sequence Type (MLST) was updated using e-BURST analysis from Rahman et al. (2014a). The resulting tree was rooted using the closely related species *Enterococcus faecalis* as outgroup.

The search for prophages was conducted with the PHAST Search Tool (Zhou et al., 2011).

### Data Availability

The annotations were deposited at DDBJ/EMBL/GenBank under the following references: PRJEB9002 for *C. maltaromaticum* ML.1.97, and PRJEB8756 for *C. maltaromaticum* 3.18. The annotations are publicly available for consultation in MicroScope^1^.

## Results and Discussion

### Prevalence of Different Carnobacterium Species in Various Ecological Niches

**FIGURE 1:**
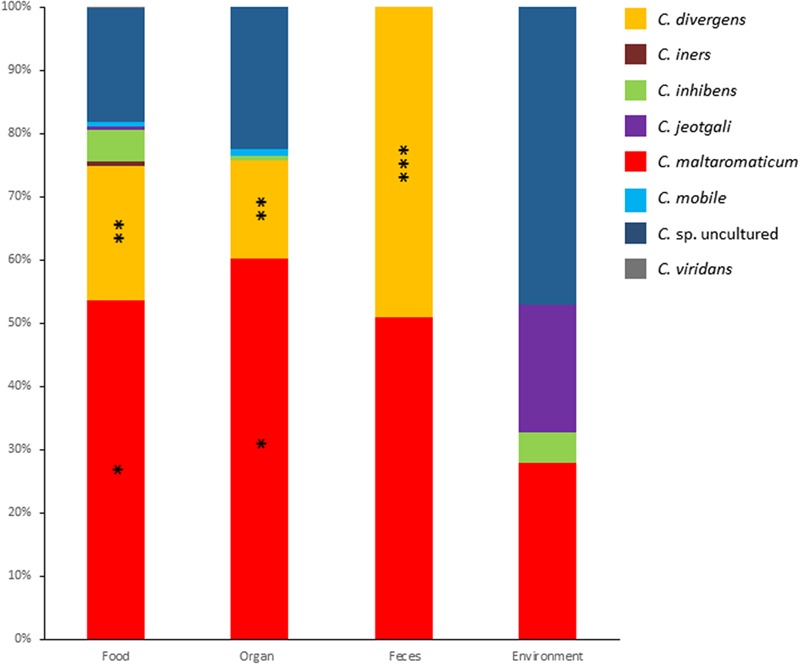
**Relative abundance of each *Carnobacterium* species obtained from 16S metagenomic analyses of 681 samples originating from food, organs, feces, and environment.** One asterisk indicate significant difference (*P* < 0.0001) compared to all other *Carnobacterium* sp, two asterisks indicate significant difference (*P* < 0.01) compared to *C. iners, C. inhibens, C. jeotgali, C. mobile*, and *C. viridans*. Three asterisks indicate significant difference (*P* < 0.0001) compared to all *Carnobacterium* sp. except *C. maltaromaticum*.

Metagenomic data for genes encoding 16S rDNA from 681 samples were analyzed with a focus on the genus *Carnobacterium*. The samples were categorized as animal-derived food, animal organs, feces, and environment. Overall, the presence of representatives of the species *C. divergens, C. iners, C. inhibens, C. jeotgali, C. maltaromaticum, C. mobile, C. viridans*, and uncultured *Carnobacterium* sp was detected and their relative abundance is presented in **Figure 1**. The most abundant species was *C. maltaromaticum* accounting for 28–60% of *Carnobacterium* reads, followed by *C. divergens* (15–49%) and *Carnobacterium* spp. from lineages that have not yet been cultured and characterized (14–47% of *Carnobacterium* reads, depending on the sample origin). *C. inhibens, C. mobile*, and *C. viridans* accounted for lesser reads. *C. jeotgali* was detected only in environmental samples. *C. viridans* was detected only in food samples whereas *C. maltaromaticum* reads were observed in the samples from all origins. Other species were detected in the samples from two or three different habitats.

The highest species diversity was observed in food and organs (dog lungs, pig nymphal nodes, pig stomach, cattle forestomach, and cattle spleen), with a maximum of eight species in food. The lowest diversity was observed in feces where only two species were recorded. In food and organs, the most abundant species were *C. maltaromaticum, C. divergens*, and uncultured *Carnobacterium* sp. The OTUs assigned to the species *C. maltaromaticum* accounted for 54% and 60% of the reads associated to the genus *Carnobacterium* in food and organs, respectively (**Figure 1**). Interestingly, in the feces only *C. maltaromaticum* and *C. divergens* sequences were detected, each accounting for about half of the reads. In contrast, *C. maltaromaticum* sequences represented 27% of the reads, while 47% of the reads were attributed to uncultured *Carnobacterium* sp. in environmental samples. *C. divergens* was not found in samples originating from the environment.

These results strongly suggest that *C. maltaromaticum* and to a lesser extend *C. divergens* are the most prevalent species of this genus in habitats associated to animals. Although animal-associated habitats differ from each other, they also share common properties: they are nutrient-rich environments, they can induce similar stresses, they are associated with a dense and diversified microbiota and carbon is mainly available in the form of polymeric biomacromolecules. It can therefore be expected that bacteria, such as *Carnobacterium* species that are associated to animal habitats, share general properties and thereby highly contrast with other *Carnobacterium* species associated with the external environment.

### Comparison of the General Genomic Features

In order to identify the adaptation factors responsible for the high ability of *C. maltaromaticum* and *C. divergens* to colonize multiple ecological niches including animal-associated habitats, a genome based analysis was conducted on nine *Carnobacterium* genomes (**Table 1**). The genome sizes of the five *C. maltaromaticum* strains were the largest, ranging from 3.33 to 3.87 Mbp; the other *Carnobacterium* genomes were smaller (2.35–2.74 Mbp), with the smallest being that of *C. inhibens* subsp. *gilichinskyi* WN1359 (**Table 2**). Accordingly, the number of predicted CDS in each genome ranged from 3,368 to 3,812 for *C. maltaromaticum*, and from 2,268 to 2,633 for the other *Carnobacterium* (**Table 2**). *C. maltaromaticum* genomes harbored a lower GC% (34.4–34.5) than other genomes (35.2–35.3).

**Table 2:** General features of *Carnobacterium* genomes.

Organisms	*C. maltaromaticum*					*C. divergens*	*C. inhibens* subsp. *gilichinskyi*	*Carnobacterium* sp.	
	LMA28	DSM20342	ATCC35586	ML.1.97	3.18	V41	WN1359	AT7	17.4
Sequence length (Mbp)	3.65	3.877	3.54	3.33	3.57	2.74	2.35	2.45	2.63
GC content (%)	34.5	34.4	34.5	34.4	34.4	35.3	35.2	35.2	35.2
Number of plasmids	3	ND	ND	ND	ND	ND	5	ND	1
Number of CDS	3,687	3,812	3,448	3,368	3,465	2,633	2,268	2,431	2,584
Number of COG	2,671	2,800	2,636	2,639	2,672	2,089	2,257	1,986	2,155
fCDS	48	12	12	49	8	15	69	12	25
Number of tRNA	59	64	61	37	59	8	75	71	67
Number of 16S-RNA	6	6	ND	ND	ND	ND	8	7	8
Prophage clusters	2^∗^ (3^∗∗^)	2 (2)	1 (4)	1	1 (5)	0 (3)	0	0 (3)	0
Scaffolds	1	5	74	229	160	32	1	69	1
Contigs	1	5	74	229	160	32	1	69	1

***^∗^** intact and **^∗∗^** region incomplete; ND, not determined.*

**FIGURE 2:**
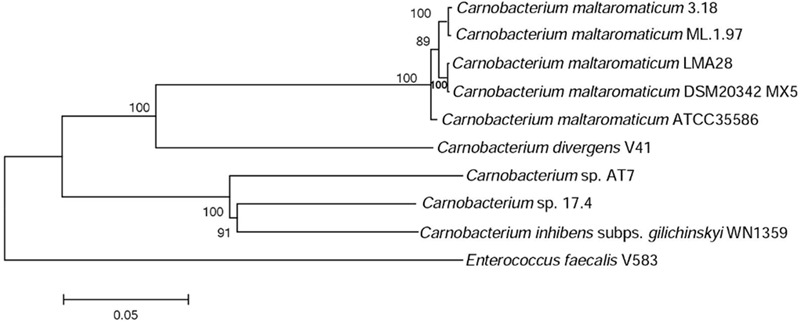
**Phylogenetic tree of the *Carnobacterium* strains subjected to comparative genome analysis.** The tree is based on the alignment of nucleic acid sequence of 10 housekeeping genes (*dnaK, gyrA, polA, lepA, dnaB, gyrB, secA, ftsZ, recG*, and *ileS*).

A phylogenetic tree based on 10 housekeeping genes was constructed. It shows that *C. maltaromaticum* and *C. divergens* on one hand, and *Carnobacterium* sp. 17.4, *Carnobacterium* sp. AT7, and *C. inhibens* subp. *gilichinskyi* WN1359 on the other hand, are closely related. They form two monophyletic taxons sharing a common ancestor, as shown by the outgroup *E. faecalis* V583 (**Figure 2**).

The pan/core genome analysis of the nine *Carnobacterium* (cut-off of 50% aa identity and 80% coverage) showed that the core genome represents 1,130 (31%) of the predicted CDS. When comparison was restricted to *C. inhibens* subsp. *gilichinskyi* WN1359, *Carnobacterium* sp. 17-4, and *Carnobacterium* sp. AT7, the core genome increased up to 1,745–1,785 (67–73%) of the predicted CDS. Similarly, when the comparison was restricted to the 5 *C. maltaromaticum* strains and *C. divergens* V41, the core genome increased up to 1,723–1,825 (68–76%) of the predicted CDS. This result is in agreement with the phylogenetic tree showing close phylogenetic proximity of *C. maltaromaticum* and *C. divergens* on the one hand, and of *C. inhibens* subsp. *gilichinskyi* WN1359, *Carnobacterium* sp. 17-4, and *Carnobacterium* sp. AT7 on the other hand.

To be more restrictive, the pan/core genome analysis was performed with a cut-off of 70% aa identity and 80% coverage. It revealed that the strains of *C. maltaromaticum* share 2,665 CDS, and each of the five strains possesses between 279 and 644 specific genes showing strain-to-strain variations.

The gene repertoire of the different genomes was then compared to search for functions that differ between strains in order to identify the genomic traits that could explain the potential adaptation of *C. maltaromaticum* and *C. divergens* to animal-linked habitats.

### CRISPR-cas and Prophages

Clustered Regularly Interspaced Short Palindromic Repeats (CRISPRs; Barrangou et al., 2007), and the CRISPR associated (cas) genes confer resistance to phage infection. The role and mechanism of the CRISPR-cas system in bacterial species have been extensively studied and indicate that the spacer sequences can be considered as a signature of past exposure to exogenous DNA. Among all *Carnobacterium* genomes, only *C. divergens* V41 possessed a CRISPR-cas system. More precisely, two loci were predicted in the genome of this strain. The presence of these genes suggests that *C. divergens* V41 may have systems acting as barriers against HGT, thereby that would limit genome expansion in this taxon.

One to four prophages and/or prophage remnants were detected in all analyzed genomes except in those of *C. inhibens* subsp. *gilichinskyi* and *Carnobacterium* sp. 17.4 (**Table 2**). Their size, ranged from 11.5 to 74.4 kbp (data not shown). More importantly, complete prophages were found only in the *C. maltaromaticum* (**Table 2**).

The genomes of all *Carnobacterium* species but one -*C. maltaromaticum*- exhibit a genome of small or relatively small sizes. This suggests that the common ancestor of *Carnobacterium* exhibited a small size and that the ancestor of the species *C. maltaromaticum* experienced a massive gain of genes. Compared to *C. divergens*, no CRISPR-Cas systems were found in the genome of *C. maltaromaticum*. CRISPR-Cas provide an adaptive immunity against foreign DNA and is considered as a barrier against horizontal transfer (Barrangou et al., 2007). The lack of such a barrier against DNA transfer could have favored the acquisition of genes in the *C. maltaromaticum* lineage. Consistently, complete prophages were found in the genomes of *C. maltaromaticum* while only remnants prophages were found in the genome of *C. divergens*. Similarly, *Lactobacillus* genomes devoid of CRISPR-Cas systems exhibited the trend of being more abundant in phage sequences (Sun et al., 2015). LAB are mainly described as evolving by massive gene loss. The lineage *C. maltaromaticum* suggests that LAB may also evolve by a massive gene gain. Consistently, other ubiquitous LAB also exhibited large genomes that could be a result of similar mechanisms (Sun et al., 2015).

### Secretome

**FIGURE 3:**
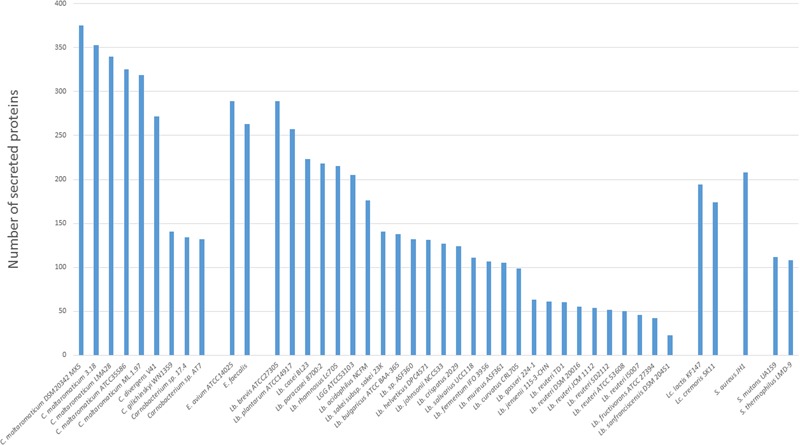
**Secretome size in the different *Carnobacterium* genomes.** Other genomes belonging to lactic acid bacteria (LAB) (*Lactococcus* and *Lactobacillus*) and other Gram-positive species (*Staphylococcus, Enterococcus*, and *Streptococcus*) available at Microscope Mage were also included.

Homologs of genes encoding the general secretion route (Sec-pathway) were present in all strains, while no homolog of the Twin-arginine translocation pathway (Tat-pathway) was found (data not shown). The secretome was therefore predicted by identifying genes suspected to encode signal peptide-containing proteins. The five *C. maltaromaticum* strains contained the largest secretome (319–375 proteins predicted to harbor a signal peptide), whereas *C. divergens* V41 presented an intermediate number (272 predicted proteins) compared to those predicted in *Carnobacterium* sp. AT7, *Carnobacterium* sp. 17.4, and *C. inhibens* subsp. *gilichinskyi* WN1359 with only 132–155 proteins containing a peptide signal. Compared to other LAB or genera that can share the same habitats, the sizes of secretomes of *C. maltaromaticum* and *C. divergens* are among the largest (**Figure 3**).

Among the COG (cluster of orthologous genes) represented in the secretome, families M (cell wall/membrane/envelop biogenesis), P (inorganic ion transport and metabolism), and R (general functional prediction only) tend to be more represented in *C. maltaromaticum* and *C. divergens* compared to the other species (Supplementary Figure S2). This suggests that extracellular functions are more diversified in *C. maltaromaticum*, and to a lesser extent in *C. divergens*, than in the other *Carnobacterium.* For instance, *C. maltaromaticum* and *C. divergens* exhibit between 22 and 35 genes in COG R, while the three other strains *Carnobacterium* sp. AT7, *Carnobacterium* sp. 17.4, and *C. inhibens* subsp. *gilichinskyi* WN1359, would have less than 10 genes encoding a signal peptide within this subclass.

Further, *C. maltaromaticum* predicted secretomes encompassed a higher number (26–30) of proteins belonging to COG family G (carbohydrate transport and metabolism) than was the case for *C. divergens* V41 (18) and other species (10–15). This is correlated with a higher content in genes encoding PTS transporters, between 62 and 68, in *C. maltaromaticum*. By contrast, the other *Carnobacterium*, including *C. divergens*, would contain less of such genes (between 27 and 43 depending on the strains). *C. maltaromaticum* strains, compared to other *Carnobacterium* species harbor a larger repertoire of PTS transporters. This characteristics is typical of ubiquitous LAB, as these transporters enable the bacteria to exploit a wide range of carbon sources (Douillard and de Vos, 2014).

More strikingly, *C. maltaromaticum* strains and *C. divergens* V41 secretomes encompassed between 116 and 155 proteins unclassified in COG families (class X Supplementary Figure S2), while this category was of minor importance (0–23) in other *Carnobacterium* species. The detailed analysis of those unclassified genes revealed that approximately half of them encode hypothetical proteins or conserved exported proteins of unknown function, and 30% encode secreted proteins associated with the cell wall. These results strongly suggest that *C. maltaromaticum* and *C. divergens* cell-surface structures differ significantly from those of *Carnobacterium* sp. AT7, *Carnobacterium* sp. 17.4, and *C. inhibens* subsp. *gilichinskyi* WN1359. Therefore, we focused on the comparison of the gene repertoire encoding such surface associated proteins.

#### Non-covalent Cell-Wall Bound Proteins

A larger set of proteins non-covalently bound to the cell wall was predicted for *C. maltaromaticum* and *C. divergens* V41 than for the other *Carnobacterium*. These proteins contain at least one LysM domain, and one WxL domain or SH3 domain (**Table 3**).

**Table 3:** Number of sortases and surface proteins.

	*C. maltaromaticum*					*C. divergens*	*C. inhibens* subsp. *gilichinskyi*	*Carnobacterium* sp.	
	LMA28	DSM20342 MX5	ATCC35586	3.18	ML.1.97	V41	WN1359	AT7	17.4
LysM	6	5	5	5	5	6	4	5	5
WxL	48	49	37	53	42	46	0	0	0
SH3	3	3	1	2	1	2	1	1	1
SDP	35	35	28	29	26	26	0	1	1
Sortase	6	8	6	6	6	5	0	1	1
Total	98	100	77	95	80	85	6	7	7

For all strains, at least one protein with an SH3 domain involved in peptidoglycan binding is predicted to be anchored to the cell wall (**Table 3** and Supplementary Table S1), and between four and six LysM proteins. Two of them are conserved in all strains, three are found only in *C. maltaromaticum* and *C. divergens* V41, and three are variable within *Carnobacterium* sp. strains (**Table 3** and Supplementary Table S1).

The signature of the 160–190 aa long WxL domain is characterized by two WxL motifs. WxL-containing proteins are non-covalently anchored proteins associated with the cell wall (Brinster et al., 2007). Strikingly, the number of WxL-containing proteins is comprised of between 37 and 53 and present only in *C. maltaromaticum* and *C. divergens*. Remarkably, no WxL-containing proteins were found to be encoded by the other *Carnobacterium* genomes. Twenty-nine genes encoding WxL proteins are common to the five *C. maltaromaticum* strains and *C. divergens* V41. Overall, 56 genes are variable within the strains (i.e., are absent in at least one strain), and three belong to the *C. maltaromaticum* core genome (**Table 3** and Supplementary Table S1). The WxL proteins belong to the cell-surface complex (Csc) protein family. The Csc protein encoding genes are typically clustered and each cluster contains at least on copy of *cscA, cscB, cscC*, and *cscD*. Similarly, *C. maltaromaticum* and *C. divergens* contain between 13 and 17 *csc* clusters. Typically, CscA contains a DUF916 domain with extracellular matrix binding ability (Galloway-Peña et al., 2015) and a C-terminal transmembrane anchor, while CscB and CscC contain WxL domains, and CscD is a small LPXTG protein (Siezen et al., 2006). Similarly, in *C. maltaromaticum* and *C. divergens*, all the WxL encoding genes are either *cscB* or *cscC* homologs, and are localized in the vicinity of at least one WxL encoding gene. These *cscB* and *cscC* homologs can also be clustered with homologs of *cscA* and *cscD*. Strikingly, the clusters can encode a high number of WxL proteins, as exemplified by the cluster BN424_324-BN424_330 which is predicted to encode six WxL proteins.

Whereas *C. maltaromaticum* and *C. divergens* are predicted to produce a high diversity of non-covalently bound proteins, only a small number of such proteins were found in *C. inhibens* subsp. *gilichinskyi* WN1359, *Carnobacterium* sp. AT7, and *Carnobacterium* sp 17. Among those, most are predicted as LysM- and SH3-containing proteins and no WxL proteins were detected (**Table 3** and Supplementary Table S1).

#### Covalently Anchored Proteins

Sortase-dependent proteins (SDP) are covalently anchored to the cell wall, and possess an LPxTG like motif at their C-terminal end. SDP nomenclature refers to proteins attached to the peptidoglycan by the sortase family of transpeptidases (Schneewind and Missiakas, 2014). Such SDP were found in *C. maltaromaticum* strains and *C. divergens* V41 whereas only one LPxTG domain protein was detected in *Carnobacterium* sp. 17.4 and *Carnobacterium* sp. AT7, and none in *C. inhibens* subsp. *gilichinskyi* WN1359 (**Table 3** and Supplementary Table S1).

Sortases decorate the surfaces of Gram-positive bacteria with diverse proteins that enable microbes to interact with their environment (Comfort and Clubb, 2004; Maresso and Schneewind, 2008). The five *C. maltaromaticum* strains and *C. divergens* V41 possess many putative sortase A and B genes, while *Carnobacterium* sp. AT7 and *Carnobacterium* sp. 17.4 harbor only one and *C. inhibens* subsp. *gilichinskyi* WN1359 possesses a pseudogene that might encode a remnant protein with similarities with sortases (**Table 3** and Supplementary Table S1).

Depending on the strains, the *C. maltaromaticum* genomes are predicted to encode 26–35 SDP, and 26 putative SDP could be predicted in *C. divergens*. Among those, 13 belong to the *C. maltaromaticum* core genome, including seven also conserved in *C. divergens.* In addition, 24 *C. maltaromaticum* SDP-encoding genes are strain specific or shared by only some of the strains. The differences between strains resulted either from the absence of homologs or the presence of 373 predicted pseudogenes. The predicted sortase-encoding gene in the genome of *Carnobacterium* sp 17.4 is located next to a collagen-binding surface protein encoding gene (ABHHv1_120049, Supplementary Table S1).

### Functions of the Surface Proteins

As *C. maltaromaticum* and, to a lesser extent, *C. divergens* presented a large panel of surface-exposed proteins compared to other *Carnobacterium* species, we searched for the putative functions of the *C. maltaromaticum* and *C. divergens* species specific proteins to illuminate the potential benefits they might provide to these two species.

#### Enzymes

**FIGURE 4:**
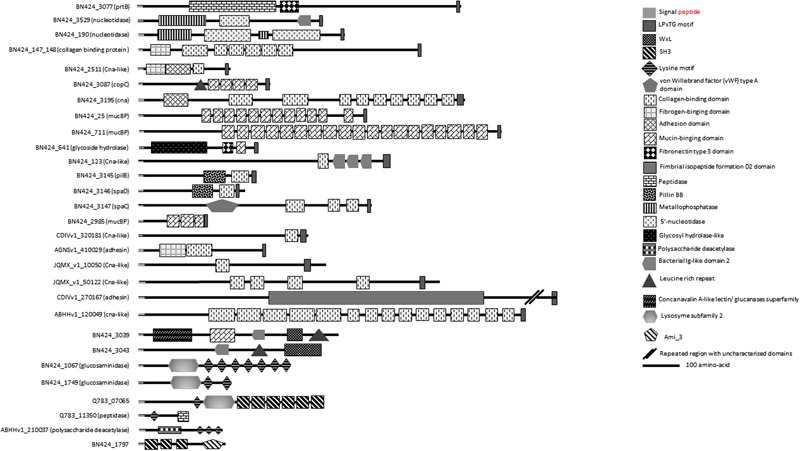
**Putative surface proteins containing predicted functional domains**.

Homologs of multidomain proteins predicted as nucleotidases/metallophosphatases as well as PrtB homologs are among the proteins conserved in *C. maltaromaticum* and *C. divergens* (**Figure 4** and Supplementary Table S1). Extracellular 5′-nucleotidase domains that catalyze dephosphorylation of exogenous adenine 5′-nucleotides to adenosine and phosphate (Bengis-Garber and Kushner, 1982) are reported as providing a key function for phosphorous regeneration in aquatic ecosystems (Ammerman and Azam, 1985).

In the dairy LAB *Lactobacillus delbrueckii* subsp. *bulgaricus*, PrtB, a cell envelope-associated protease (CEP) has been shown to degrade casein into peptides (Siezen, 1999). Peptides and aas are subsequently internalized and peptides are further hydrolyzed by intracellular peptidases into small peptides and free aa (Savijoki et al., 2006). The analysis of the genomes of *C. maltaromaticum* and *C. divergens* revealed the conserved presence of oligopeptides transporter systems OppABCDF and DtpT, as well as intracellular peptidases (Supplementary Table S1). Interestingly, homologs of the Opp system and of intracellular peptidases were also found in *Carnobacterium* sp. 17.4, *Carnobacterium* sp. AT7, and *C. inhibens* subsp. *gilichinskyi* WN1359 but no homologs of CEP and no DtpT. In the dairy environment, CEP are believed to confer a selective advantage by allowing bacteria to exploit the aa from milk casein (Kunji et al., 1996; Christensen et al., 1999; Doeven et al., 2005; Savijoki et al., 2006). All these data strongly suggest that among *Carnobacterium* only *C. maltaromaticum* and *C. divergens* are indeed able to exploit aa from the proteins present in their environments. The presence of PrtB would be a selective advantage for these two species in protein-rich environments such as food.

Among the SDP proteins present in several *C. maltaromaticum* strains we noticed a CDS predicted as harboring a glycoside hydrolase activity (BN424_641, **Figure 4** and Supplementary Table S1). This multidomain protein also contain a fibronectin type III-like module of unknown function, which is usually associated with glycoside hydrolase domains (Alahuhta et al., 2010). Such enzymatic activity could allow *C. maltaromaticum* to degrade extracellular carbohydrate polymers. Interestingly, this protein is also predicted to contain a mucin-binding domain. Mucin is the major component of the intestinal mucus. It is tempting to speculate that the putative mucin-binding glycoside hydrolase of *C. maltaromaticum* would hydrolyze mucin glycan moieties as has been described for some gut bacteria (Tailford et al., 2015).

#### Nutrient Uptake: Heme Compounds

**FIGURE 5:**
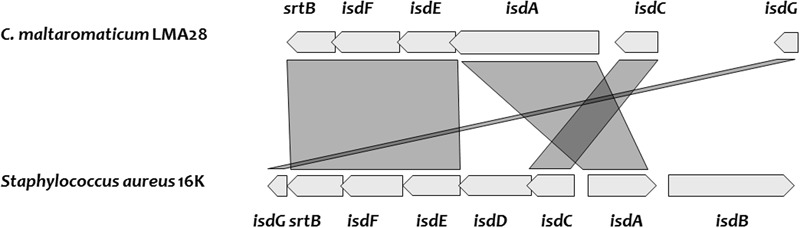
**Genes encoding heme uptake system, cluster organization, and synteny between *C. maltaromaticum* LMA28 and *Staphylococcus aureus* 16K**.

Two SDP, predicted as heme-binding proteins in *C. maltaromaticum* and *C. divergens*, are homologs of IsdA and IsdC (**Figure 5** and Supplementary Table S1). The Isd system in *Staphylococcus aureus* enables to utilize different sources of heme: free heme, heme bound to free hemoglobin, and heme bound to hemoglobin interacting with haptoglobin. The Isd ABC-transport system in *S. aureus* is encoded by *isdABCDEFGH;* IsdH, IsdB, and IsdA acting as cell wall anchored receptor proteins: IsdH is the primary haptoglobin-hemoglobin receptor, IsdB the primary hemoglobin receptor, and IsdA can bind free heme or accept heme from IsdB or IsdH. Heme is subsequently transferred from IsdA to IsdC, another cell wall protein and then to a membrane associated transporter, formed by IsdD, IsdE, and IsdF. After internalization, heme is taken up by the heme degrading monooxygenase IsdG that releases the resulting iron in the cytoplasm (Choby and Skaar, 2016). At first glance, among the possible protein candidates able to bind heme from the environment, *C. maltaromaticum* and *C. divergens* would only have IsdA as no ortholog of IsdB and IsdH were found. This would suggest that these two *Carnobacterium* species would primary be able to use free heme as iron source and not heme bound to proteins. However, the IsdA homolog in *C. maltaromaticum* is predicted to contain four near-iron transporter (NEAT) domains while IsdA from *S. aureus* contains only two. NEAT domains bind heme compounds or proteins containing heme compound (Gaudin et al., 2011; Balderas et al., 2012). It could therefore be speculated that the presence of two additional NEAT domains in the *C. maltaromaticum* IsdA homolog might compensate the absence of IsdB.

*Carnobacterium divergens* and *C. maltaromaticum* do not exhibit any IsdD homolog. However, we found two *fhuC* homologs which encode an ATP-binding component of ABC transporter as well. These FhuC homologs could play the equivalent role of IsdD and thus build a functional ABC transporter with the permease encoded by the IsdF homolog. In *C. divergens* V41, one *fhuC* is localized in the vicinity of the putative *isd* genes, while in the *C. maltaromaticum* strains, this homolog is localized elsewhere in the genome. In addition, all the analyzed genomes of *Carnobacterium* contain an *isdG* homolog and would be able to release iron after heme import in the cytoplasm. The *isdA* homolog of *C. maltaromaticum* ML1.97 and 3.18 appear as a pseudogene, indicating that this system is not fully functional in these two strains. Overall, these analyses suggest *C. divergens* V41 and several strains if not all of *C. maltaromaticum* would be able to use extracellular heme, for respiration (see below) and/or as an iron source.

#### Microbial Adhesion

The previous analysis of the genome of *C. maltaromaticum* LMA28 reported the presence of putative cell-surface adhesins (Rahman et al., 2014b). Comparison of *Carnobacterium* genomes revealed that *C. maltaromaticum* strains and *C. divergens* V41 may produce several surface proteins predicted to contain domains reminiscent of adhesion function: collagen-binding, mucBP, Leucine-Rich Repeat (LRR).

Among those, 10 LPxTG proteins are predicted to have a collagen-binding domain (Supplementary Table S1 and **Figure 4**). Functional domains are annotated collagen-binding protein, Cna, or bacterial adhesin. Collagen-binding proteins found in *S. aureus* (Elasri et al., 2002) and *Listeria monocytogenes* (Bierne and Cossart, 2007) are suggested to participate in the infection process. However, proteins putatively involved in adhesion to collagen and mucin have also been reported to be important for the probiotic properties of LAB as shown in *Lactobacillus plantarum* WCFS1 (Boekhorst et al., 2006b). It is therefore difficult to predict if the binding capacity of such proteins in *Carnobacterium* can be considered as beneficial or not.

Three LPxTG proteins (**Figure 4** and Supplementary Table S2) contain a MucBP (mucin-binding protein) domain. MucBP domains allow adhesion to mucus material (Lukić et al., 2012). *Lactobacillales* and *Listeria* species can possess between 1 and 14 MucBP-containing proteins (Boekhorst et al., 2006a; Bierne and Cossart, 2007).

In *Enterococcus faecium*, the WxL protein SwpA and the protein DufA, which contains a DUF916 domain and is encoded by a gene that belong to a *csc* cluster, are collagen and fibronectin-binding proteins (Galloway-Peña et al., 2015). Similarly, it can be hypothesized that at least some WxL and DUF916 likely encoded by *C. divergens* and *C. maltaromaticum* might exhibit similar adhesion properties. In addition, two LRR domains were found in some strains of *C. maltaromaticum* holding a WxL anchorage domain (**Figure 4** and Supplementary Table S2). These domains are involved in protein–protein interactions. They are described to be associated with domains exhibiting an Ig-like (immunoglobulin) fold, the function of which is thought to facilitate the presentation of the adjacent LRR domain (Bierne and Cossart, 2007). Accordingly, near the LRR domain of the putative *C. maltaromaticum* LMA28 surface protein BN424_3043, an Ig-like domain was found at the C-terminal end of the LRR region (**Figure 4** and Supplementary Table S2). In *L. monocytogenes*, internalins associated to virulence are LRR-containing proteins. LRR-containing proteins are rather uncommon in *Lactobacillus*.

The presence of such putative adhesins suggests that *C. maltaromaticum* and *C. divergens* exhibit the ability to adhere to intestinal mucosa and extracellular matrices of animals. Overall, 19 putative adhesins were predicted from the genomes of *C. maltaromaticum* and *C. divergens* and absent from the other *Carnobacterium* species. All these proteins are SDP except two LRR-containing proteins which exhibit a WxL binding domain. Of these 19 proteins, two are conserved in all *C. maltaromaticum* strains and absent in other *Carnobacterium*: a putative collagen-binding SDP and a mucin-binding protein.

**FIGURE 6:**
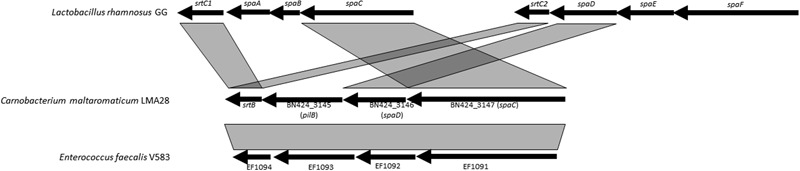
**Genetic organization and synteny of pili synthesis genes between *C. maltaromaticum* LMA28, *Lactobacillus rhamnosus* GG, and *Enterococcus faecalis* V583**.

Genes encoding the pili proteins previously described for *C. maltaromaticum* LMA28 (Rahman et al., 2014b), were found only in one other *C. maltaromaticum* strain (DSM20342 MX5). Both strains belong to clonal complex CC1, which is suspected to be a lineage well-adapted to the dairy environment. Pili were described as surface components promoting adhesion to dairy matrix in the probiotic strain *L. rhamnosus* GG and thereby they might contribute to confer an advantage in dairy products (Burgain et al., 2014). In general in Gram-positive bacteria, two or three genes encoding the pilus subunits are organized into an operon, along with at least one sortase gene (Mandlik et al., 2008; Proft and Baker, 2009). The closest homologs of such *C. maltaromaticum* genes were found in *E. faecalis*, with also a highly similar genetic organization (**Figure 6**). However, pilin gene organization is different between *C. maltaromaticum* and *L. rhamnosus* GG suggesting that the genetic structures of pili loci in *C. maltaromaticum, E. faecalis* V583, and *L. rhamnosus* GG are the result of different gene rearrangements.

Overall, these comparative genomic analyses suggest that all *C. maltaromaticum* strains and *C. divergens* might have adhesive properties and that strains might exhibit differences in this regard.

Further, these analyses demonstrated striking differences between the group *C. maltaromaticum*/*C. divergens* and the other *Carnobacterium* spp. The secretome and more specifically the cell-surface proteome of *C. maltaromaticum* and *C. divergens* are large, the one of *C. maltaromaticum* being the largest described for LAB as previously noticed by Sun et al. (2015). Conversely the secretome of *C. inhibens* subsp. *gilichinskyi* WN1359, *Carnobacterium* sp. AT7, and *Carnobacterium* sp. 17.4 is the smallest among LAB. The detailed analysis of the functions provided by such large secretome supports a prediction that the cell-surface proteome would confer the ability to hydrolyze and to adhere to biomacromolecules as well as to capture biomolecules (heme compounds). The cell surface of *C. maltaromaticum* and *C. divergens* might closely interact with animal environments and use these nutrient-rich substrates. In addition, *C. maltaromaticum* would likely exhibit a larger repertoire of hydrolytic enzymes and adhesins that may enable *C. maltaromaticum* to adapt to multiple habitats. By contrast, the other *Carnobacterium* lack such a cell-surface proteome and would therefore be expected to be less able to colonize animal-linked habitats. The size of the secretome is highly variable in LAB. Interestingly, all known specialized LAB *Streptococcus thermophilus, L. iners, L. reuteri, L. sanfranciscensis*, and *L. fructivorans* are characterized by a small secretome, suggesting that colonizing one specific niche, such as dairy, vagina, sourdough, or the GI tract, respectively, does not require a large secretome. Consistently, the known generalist LAB such as *L. rhamnosus, L. plantarum* (Martino et al., 2016), and some *Enterococcus* spp. exhibit a large secretome. We propose that there is an intimate relationship between the secretome size and the ability of LAB to colonize diverse habitats. According to this hypothesis, the bigger a secretome, the higher the capacity to colonize multiple environments.

### Respiration

*Carnobacterium maltaromaticum* is unable to synthesize heme and exhibits better growth efficiency in the presence of hematin, suggesting that heme is used by *C. maltaromaticum* to respire oxygen. Consistent with this hypothesis, *C. maltaromaticum* has been reported to produce cytochrome b and d types when grown aerobically with hematin (Meisel et al., 1994). The gene repertoire of *C. maltaromaticum* suggests the ability to produce a functional respiratory chain. Indeed, the electron donor-encoding genes, *noxB* and *ndh*, and those required for the synthesis of the electron shuttle menaquinone (*yhdB, menE, menB, menD, menF, ispA, ispB*, and *menA*) were present. Further, *cydABCD*, encoding the heme-dependent cytochrome quinol oxidase that performs the final electron transfer to the acceptor oxygen (Lechardeur et al., 2011), were also found. These genes were present in all *C. maltaromaticum* strains and in *C. divergens*, except in *C. maltaromaticum* ML.1.97 whose *menF* appears to be a pseudogene. Therefore, this last strain might require the presence of quinone in the environment to respire, as reported for *Streptococcus agalactiae* (Rezaïki et al., 2008). It seems therefore that the components of the cell wall proteome involved in extracellular heme utilization may also contribute to oxygen respiration in *C. maltaromaticum* and *C. divergens*.

By contrast, only *menA, noxA/noxB*, and *ispB* were found in *Carnobacterium* sp. AT7, *Carnobacterium* sp. 17.4, and *Carnobacterium inhibens* subsp. *gilichinskyi* WN1359 strongly suggesting that these three bacteria do not possess any functional respiratory chain.

### Adaptation to the GI Tract

Bacteria have to deal with several stresses in order to survive in the GI tract, including the immune system and bile. All strains of *C. maltaromaticum* and *C. divergens* contain genes conferring resistance to some components of the immune system. Indeed, homologs were found for *mprF, dltABCD, asnH/asnB, oatA*, and *pgdA* (Supplementary Table S2). The genes *mprF* and *dltABCD* are involved in phospholipid lysinylation and teichoic acid D-alanylation, respectively, and thus confer resistance to antimicrobial peptides of the innate immune system by protecting the cell wall. The genes *oatA* and *pgdA* confer to peptidoglycan a high resistance to lysozyme, another component of the innate immune system, by introducing *O*-acetylation and *N*-deacetylation, respectively. Importantly, no *mprF, dltABCD, asnH/asnB, oatA*, and *pgdA* homologs were found in the genomes of *Carnobacterium* sp. AT7, *Carnobacterium* sp. 17.4, and *Carnobacterium inhibens* subsp. *gilichinskyi* WN1359.

Bile salt hydrolase encoding genes were conserved in all strains of *C. maltaromaticum* while none were found in other Carnobacteria including *C. divergens* V41 (Supplementary Table S2).

*Carnobacterium maltaromaticum* and *C. divergens* are thus highly contrasting from the three other *Carnobacterium* taxons by their content of genes described in *Lactobacillus* as key factor for survival in the GI tract of animals (Kleerebezem et al., 2010). Indeed, while *C. maltaromaticum* and *C. divergens* possess genes putatively conferring resistance against the innate immune system, almost none of these homologs where found in *Carnobacterium* sp. AT7, *Carnobacterium* sp. 17.4, and *C. inhibens* subsp. *gilichinskyi* WN1359. This might explain why the only two species identified in feces samples are *C. maltaromaticum* and *C. divergens.* However, surprisingly, genes allowing resistance to bile were only found in *C. maltaromaticum* and not in *C. divergens.* Yet the ability to hydrolyze bile is described as a key factor for colonization of the gut (Kleerebezem et al., 2010; Seedorf et al., 2014). Indeed *C. maltaromaticum* LMA28, a cheese isolate that possesses such genes, is able to survive during the gastrointestinal transit in a mouse model (Rahman et al., 2014b; Sun et al., 2015). However, such ability has not yet been tested in *C. divergens* V41. Whether these two species differ in their ability to deal with bile and the ecological consequences it has on GI tract survival deserves further investigation.

## Author Contributions

Performed 16S meta-barcoding: BT and GD. Performed whole genome sequencing and assembly: JL, MH, and SS. Comparative genome analyses: CI, CC-G, FB, A-MR-J, MZ, and BR. Wrote the manuscript: CI, CC-G, BT, MZ, BR, JL, CM, FB, and A-MR-J. Coordinated the study: FB.

## Conflict of Interest Statement

The authors declare that the research was conducted in the absence of any commercial or financial relationships that could be construed as a potential conflict of interest.

## Footnotes

1 http://www.genoscope.cns.fr/agc/microscope/home/
