# Supplementary material for: Comparative Genomic Analysis Reveals Ecological Differentiation in the Genus Carnobacterium
Source: Front Microbiol. 2017 Mar 8;8:357. doi: 10.3389/fmicb.2017.00357 (PMC5341603; doi:10.3389/fmicb.2017.00357)
Supplement: Supplementary file 3 [file Presentation_1.PPTX]

## Slide 1
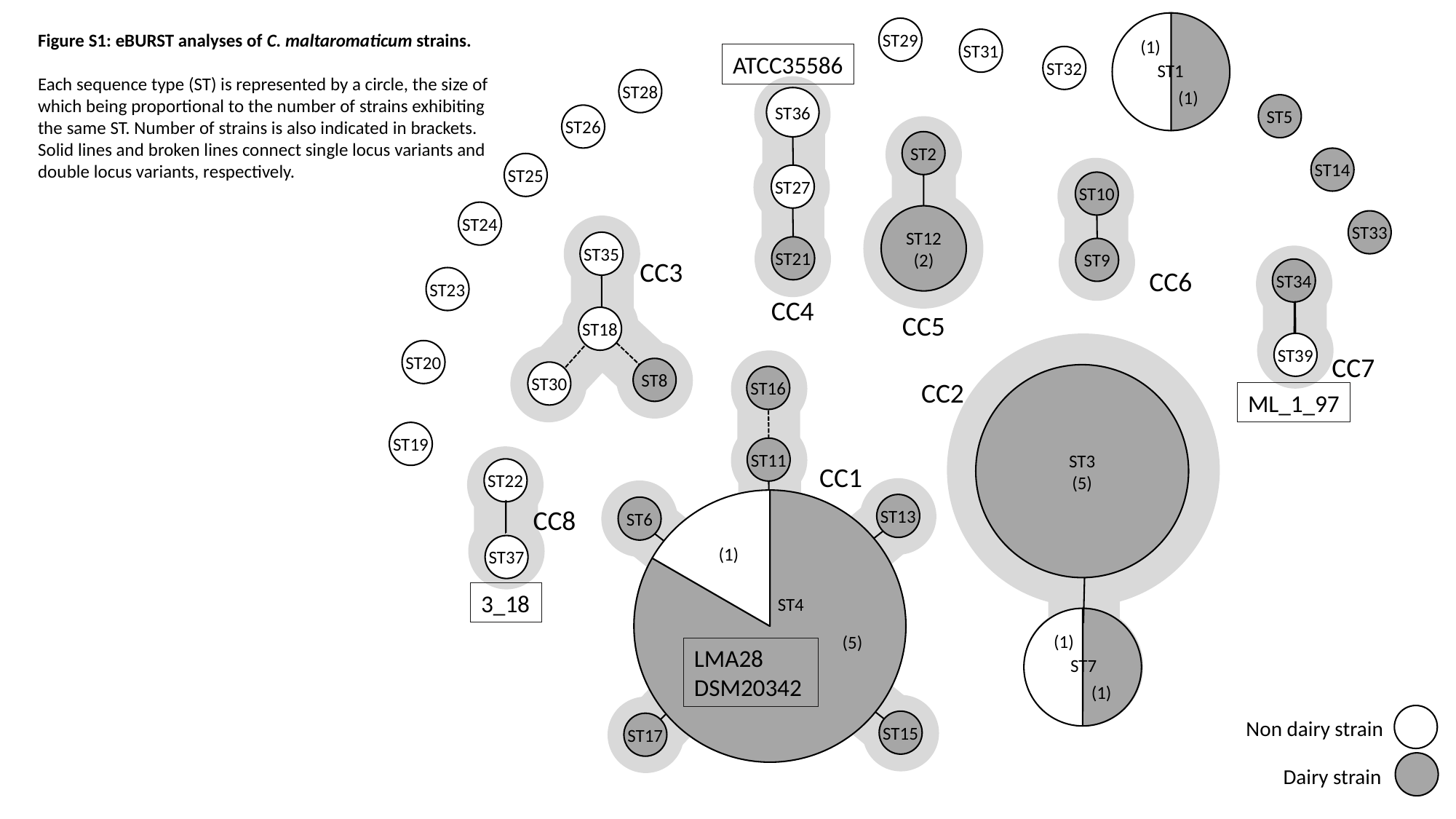

### Chart
| Category | Ventes |
|---|---|
| 1er trim. | 1.0 |
| 2e trim. | 1.0 |(1)
ST1
(1)
ST29
ST31
ATCC35586
ST32
ST28
ATCC
ST36
ST5
ST26
ST2
ST12
(2)
CC5
ST14
ST25
ST27
ST21
CC4
ST10
ST9
CC6
ST24
ST33
ST35
CC3
ST34
ST23
ST18
ST39
ST20
CC7
ST8
ST30
ST3
(5)
ST16
CC2
ML_1_97
ST19
ST11
CC1
ST22
### Chart
| Category | Ventes |
|---|---|
| 1er trim. | 5.0 |
| 2e trim. | 1.0 |(1)
ST4
(5)
ST13
ST6
CC8
ST37
3_18
### Chart
| Category | Ventes |
|---|---|
| 1er trim. | 1.0 |
| 2e trim. | 1.0 |
(1)
LMA28
DSM20342
ST7
(1)
Non dairy strain
ST15
ST17
Dairy strain
Figure S1: eBURST analyses of C. maltaromaticum strains.
Each sequence type (ST) is represented by a circle, the size of which being proportional to the number of strains exhibiting the same ST. Number of strains is also indicated in brackets. Solid lines and broken lines connect single locus variants and double locus variants, respectively.

## Slide 2
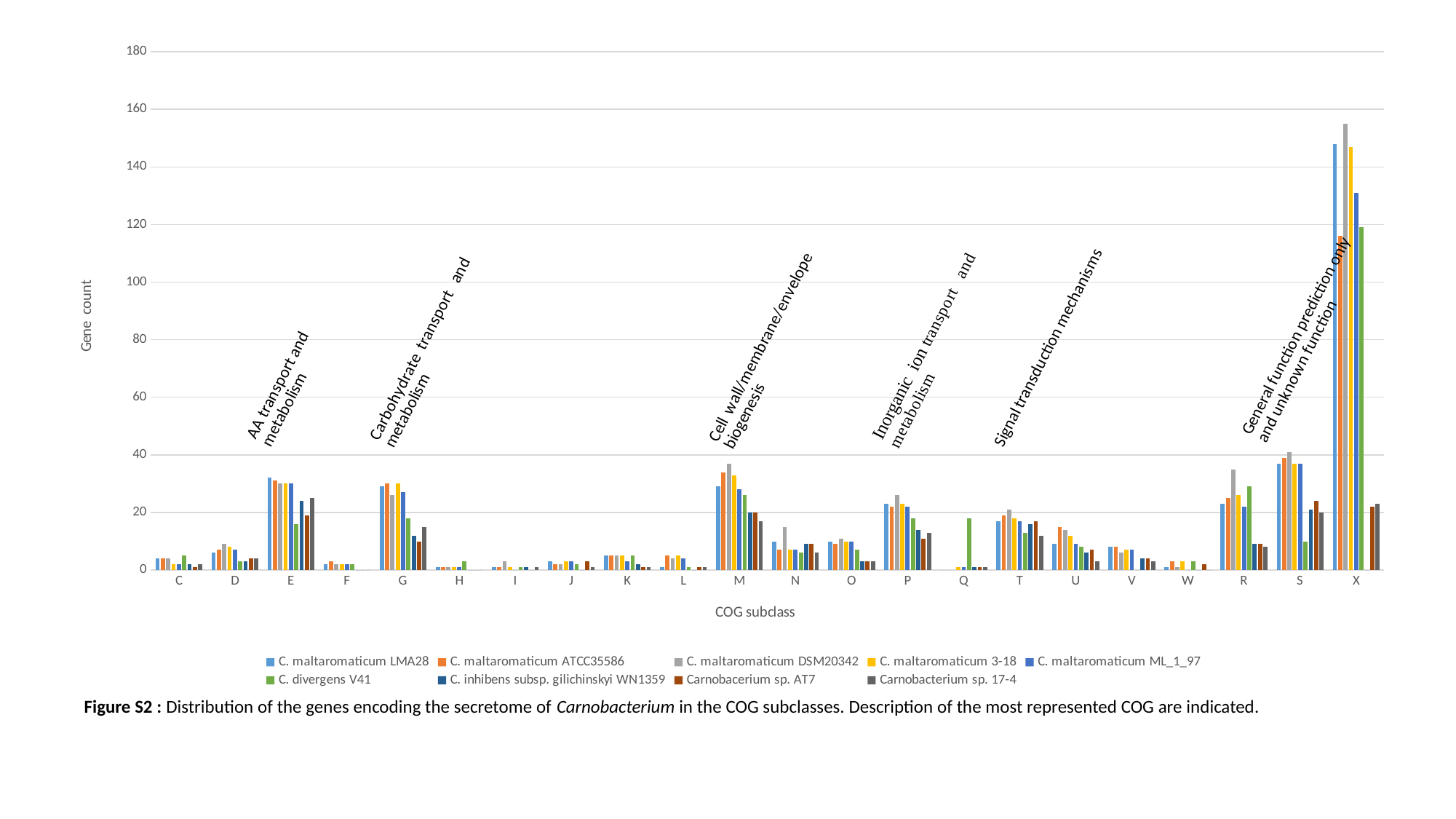

### Chart
| Category | C. maltaromaticum LMA28 | C. maltaromaticum ATCC35586 | C. maltaromaticum DSM20342 | C. maltaromaticum 3-18 | C. maltaromaticum ML_1_97 | C. divergens V41 | C. inhibens subsp. gilichinskyi WN1359 | Carnobacerium sp. AT7 | Carnobacterium sp. 17-4 |
|---|---|---|---|---|---|---|---|---|---|
| C | 4.0 | 4.0 | 4.0 | 2.0 | 2.0 | 5.0 | 2.0 | 1.0 | 2.0 |
| D | 6.0 | 7.0 | 9.0 | 8.0 | 7.0 | 3.0 | 3.0 | 4.0 | 4.0 |
| E | 32.0 | 31.0 | 30.0 | 30.0 | 30.0 | 16.0 | 24.0 | 19.0 | 25.0 |
| F | 2.0 | 3.0 | 2.0 | 2.0 | 2.0 | 2.0 | 0.0 | 0.0 | 0.0 |
| G | 29.0 | 30.0 | 26.0 | 30.0 | 27.0 | 18.0 | 12.0 | 10.0 | 15.0 |
| H | 1.0 | 1.0 | 1.0 | 1.0 | 1.0 | 3.0 | 0.0 | 0.0 | 0.0 |
| I | 1.0 | 1.0 | 3.0 | 1.0 | 0.0 | 1.0 | 1.0 | 0.0 | 1.0 |
| J | 3.0 | 2.0 | 2.0 | 3.0 | 3.0 | 2.0 | 0.0 | 3.0 | 1.0 |
| K | 5.0 | 5.0 | 5.0 | 5.0 | 3.0 | 5.0 | 2.0 | 1.0 | 1.0 |
| L | 1.0 | 5.0 | 4.0 | 5.0 | 4.0 | 1.0 | 0.0 | 1.0 | 1.0 |
| M | 29.0 | 34.0 | 37.0 | 33.0 | 28.0 | 26.0 | 20.0 | 20.0 | 17.0 |
| N | 10.0 | 7.0 | 15.0 | 7.0 | 7.0 | 6.0 | 9.0 | 9.0 | 6.0 |
| O | 10.0 | 9.0 | 11.0 | 10.0 | 10.0 | 7.0 | 3.0 | 3.0 | 3.0 |
| P | 23.0 | 22.0 | 26.0 | 23.0 | 22.0 | 18.0 | 14.0 | 11.0 | 13.0 |
| Q | 0.0 | 0.0 | 0.0 | 1.0 | 1.0 | 18.0 | 1.0 | 1.0 | 1.0 |
| T | 17.0 | 19.0 | 21.0 | 18.0 | 17.0 | 13.0 | 16.0 | 17.0 | 12.0 |
| U | 9.0 | 15.0 | 14.0 | 12.0 | 9.0 | 8.0 | 6.0 | 7.0 | 3.0 |
| V | 8.0 | 8.0 | 6.0 | 7.0 | 7.0 | 0.0 | 4.0 | 4.0 | 3.0 |
| W | 1.0 | 3.0 | 1.0 | 3.0 | 0.0 | 3.0 | 0.0 | 2.0 | 0.0 |
| R | 23.0 | 25.0 | 35.0 | 26.0 | 22.0 | 29.0 | 9.0 | 9.0 | 8.0 |
| S | 37.0 | 39.0 | 41.0 | 37.0 | 37.0 | 10.0 | 21.0 | 24.0 | 20.0 |
| X | 148.0 | 116.0 | 155.0 | 147.0 | 131.0 | 119.0 | 0.0 | 22.0 | 23.0 |Figure S2 : Distribution of the genes encoding the secretome of Carnobacterium in the COG subclasses. Description of the most represented COG are indicated.
